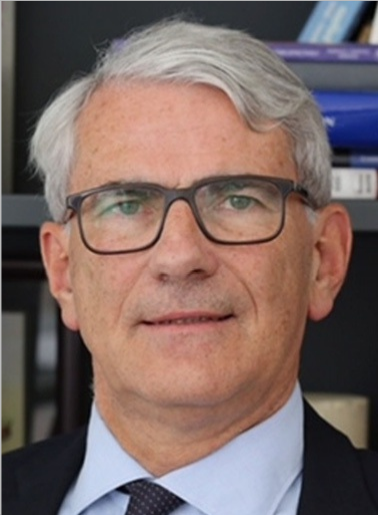# Formation and Functioning of Bimetallic Nanocatalysts: The Power of X‐ray Probes

**DOI:** 10.1002/anie.201902859

**Published:** 2019-07-24

**Authors:** Matthias Filez, Evgeniy A. Redekop, Jolien Dendooven, Ranjith K. Ramachandran, Eduardo Solano, Unni Olsbye, Bert M. Weckhuysen, Vladimir V. Galvita, Hilde Poelman, Christophe Detavernier, Guy B. Marin

**Affiliations:** ^1^ Laboratory for Chemical Technology Ghent University Technologiepark 125 9052 Ghent Belgium; ^2^ Inorganic Chemistry and Catalysis group Utrecht University Universiteitsweg 99 3584CG Utrecht The Netherlands; ^3^ Centre for Materials Science and Nanotechnology (SMN) Department of Chemistry University of Oslo P.O box 1126 Blindern C0318 Oslo Norway; ^4^ Conformal Coatings of Nanomaterials group Ghent University Krijgslaan 281/S1 9000 Ghent Belgium; ^5^ NCD-SWEET beamline ALBA synchrotron light source Carrer de la Llum 2–26 08290, Cerdanyola del Vallès Barcelona Spain

**Keywords:** bimetallic nanocatalysts, deactivation, catalyst formation, X-ray characterization

## Abstract

Bimetallic nanocatalysts are key enablers of current chemical technologies, including car exhaust converters and fuel cells, and play a crucial role in industry to promote a wide range of chemical reactions. However, owing to significant characterization challenges, insights in the dynamic phenomena that shape and change the working state of the catalyst await further refinement. Herein, we discuss the atomic‐scale processes leading to mono‐ and bimetallic nanoparticle formation and highlight the dynamics and kinetics of lifetime changes in bimetallic catalysts with showcase examples for Pt‐based systems. We discuss how in situ and operando X‐ray spectroscopy, scattering, and diffraction can be used as a complementary toolbox to interrogate the working principles of today's and tomorrow's bimetallic nanocatalysts.

## 1 Introduction

Bimetallic nanoparticle (NP) catalysts display extraordinary physicochemical properties compared to their monometallic counterparts.^1^, ^2^, ^3^, ^4^ When initially introduced by Sinfelt et al., Ni‐Cu, Ru‐Cu, and Os‐Cu were found to reduce undesired C−C activation for hydrogenolysis compared to monometallic Ni, Ru, and Os, while maintaining their C−H activation abilities for dehydrogenation.^5^ This early discovery triggered the exploration and widespread use of bimetallic nanocatalysts, including Pt‐Pd, Pt‐Ru, Pt‐Ir, Pt‐Re alloys, to induce selectivity shifts towards desired reaction products.^6^, ^7^, ^8^, ^9^ Today, bimetallic NPs possess recognized abilities to promote reforming, hydrogenolysis, (de)hydrogenation, and oxidation reactions.^10^ Recently, attention has been invested in the development of bimetallic catalysts for upgrading biomass to fuels and chemicals,^10^, ^11^ the production of hydrogen,^12^ as well as in the use of earth‐abundant transition (bi)metallic nanocatalysts.^13^ Bimetallic NP electrocatalysis^14^ is another application area, which however lies beyond the scope of this Minireview.

The origin of the performance shift in bimetallic compared to monometallic nanocatalysts is mainly attributed to electronic (ligand) and geometric (ensemble) effects.^15^ By alloying a transition metal with a donor metal, the d‐band center shifts to lower energies, thereby reducing the adsorbate–metal bond strength, which alters its selectivity. The geometric effect results from the decrease of the active metal ensemble size at the surface of a bimetallic NP owing to the presence of alloying metals. Such decrease can result in (partial) inhibition of structure sensitive reactions for which active metal islands are required, and hence forms a tool to steer the catalyst selectivity.

Ideally, an atomically tailored bimetallic nanocatalyst can be fabricated with meticulously selected NP shape, size, composition, and stability to yield maximal activity and full selectivity towards target reaction products. Whilst increasingly realistic catalyst screening methods have been developed by computational approaches,^16^ experimental characterization of the bimetallic nanocatalyst is equally required. Particularly, charting all possible active sites in a single bimetallic NP and probing their abundance and chemical and structural nature during catalyst formation and functioning will provide input for the pursued rational design.^17^ X‐ray‐based characterization is ideal for monitoring the electronic and structural state of (bi)metallic nanocatalysts. In view of the decades‐long development of X‐ray tools and the advent of X‐ray sources with unprecedented opportunities, this Minireview provides a brief overview of the current X‐ray toolbox for nanocatalyst characterization and its application under relevant conditions.

## 2 Bimetallic Nanocatalyst and its Complexity

**Figure 1:**
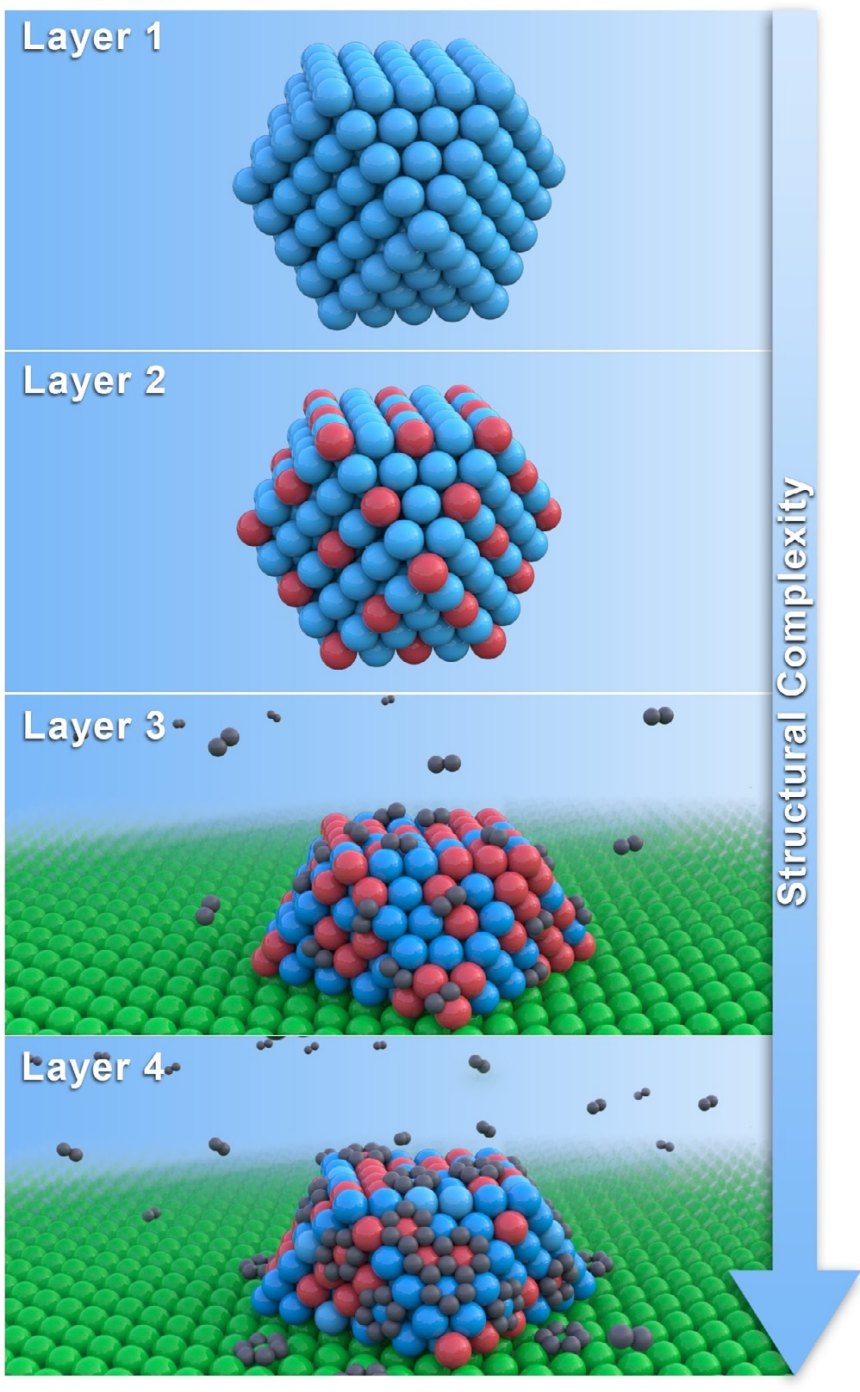
Layers of complexity in bimetallic nanocatalysts. Layer 1: intracrystal site heterogeneities at corners, edges, and facets. Layer 2: change in active site number and nature by second metal alloying. Layer 3: NP changes owing to external ligands. Layer 4: active site modification and poisoning during reaction.

(Bi)metallic catalysts were initially regarded as static entities. However, the past decades have brought firm understanding that (supported) metal catalysts are dynamic nanomaterials with evolving properties over the catalyst lifetime. For example, metal nanocatalysts in action can reversibly switch between work and sleep mode, or communicate within and between single NPs, reminiscent of living organisms.^18^, ^19^ Understanding the intricate nanoscale phenomena which underlie catalyst formation, functioning, and aging is a formidable challenge owing to their complexity. To decipher the observed phenomena in greater detail, defining four layers of complexity aids in deconvoluting the structural complexity of bimetallic nanocatalysts. These four layers are shown in Figure 1.

### 2.1 Layer 1: Active Site Heterogeneity

A NP is a solid ensemble of atoms with finite dimensions, typically exposing low‐indexed facets to minimize the Gibbs free energy. The NP surface contains low‐coordinated atoms (the active sites) situated at corners, edges, kinks, and terraces with different reactivity.^20^ Such heterogeneity in the nature of the active sites introduces the first layer of complexity in structure–performance relationships, even for the most simple case of a monometallic NP. Cuenya et al. showed that differently‐shaped 1 nm Pt NPs have lower onset temperature for 2‐propanol partial oxidation owing to a higher degree of Pt undercoordination.^21^

### 2.2 Layer 2: Second Metal

The second layer of complexity arises owing to the change in the active site number and nature upon introduction of a second metal to the NP. The active site distribution on the bimetallic NP surface depends on its mixing configuration, ranging from core–shell to ordered and randomly homogeneous alloys.^11^ The mixing enthalpy and entropy determine the intimacy of mixing and degree of alloy ordering, respectively, and thereby the active site number and nature. Typically, the metal with lowest sublimation temperature will segregate to the NP surface (under specific conditions), affecting the active site number.^5^ Notably, nanoalloy formation can lead to NP shape and size changes compared to their monometallic analogues.

### 2.3 Layer 3: External Ligands: Support and Environment

The third layer of complexity arises from the structural and chemical transformation of the bimetallic NP owing to the reaction environment, either gas or liquid, and the support. These interactions provide external ligands to the bare bimetallic NP, which can strongly alter its shape, size, composition, strain, and electronic properties, and thereby the catalytic performance. For example, Mager‐Maury et al.^22^ and Tao et al.^23^ demonstrated that H_2_ and CO change the shape and size of Pt NPs, respectively. Frenkel et al.^24^ provided evidence of gas‐ and support‐induced lattice strain in Pt NPs. The degree of charge transfer between the support and the NP (Schwabb effect) can affect the NP electron density, as shown by Lykhach et al.^25^ for Pt/CeO_2_.

### 2.4 Layer 4: Active‐Site Modification and Poisoning

Supported bimetallic NPs under reaction conditions are prone to reaction‐induced active site modification and poisoning. The former changes the nature of the metal site and includes the incorporation of subsurface H and C in Pd hydrogenation catalysts or oxidation state changes at the NP surface.^26^, ^27^ These modifications can improve or inhibit the catalytic activity. In contrast, poisoning hinders further reaction and reduces the number of available active sites. Most common are active site blockage by hydrocarbon‐derived CH_x_ fragments or irreversible adsorption by CO or S.^28^, ^29^ NP sintering can be classified under poisoning as well, considering it blocks the original metal site by the active metal itself, causing an irreversible decrease in the number of available sites.

## 3 The X‐ray Toolbox

**Figure 2:**
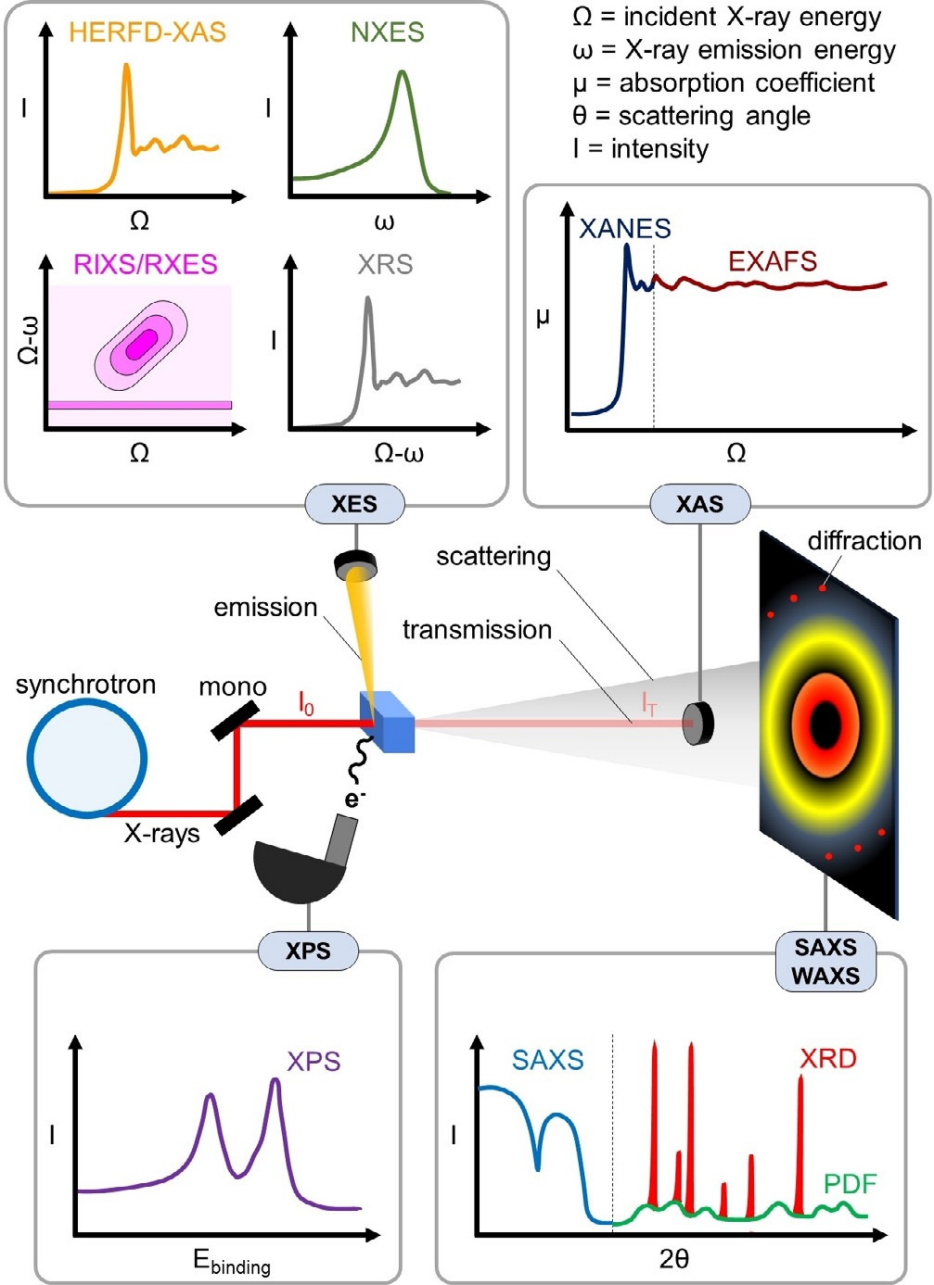
Overview of synchrotron‐based X‐ray tools. Monochromatic X‐rays irradiate the sample, leading to absorption, emission, scattering, diffraction, and photoelectron emission. XES, grouping HERFD‐XAS, non‐resonant XES (NXES), resonant XES (RXES) or resonant inelastic X‐ray scattering (RIXS), and X‐ray Raman scattering (XRS); XAS, grouping XANES and EXAFS; SAXS‐WAXS, grouping SAXS, XRD and PDF; XPS.

**Figure 3:**
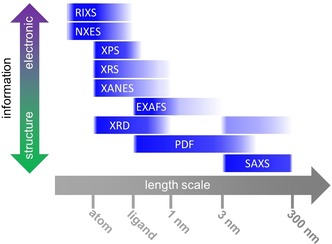
Overview of structural and electronic information content that X‐ray tools provide on functional nanomaterials over different length scales.

X‐ray sources have revolutionized materials characterization at an unprecedented pace. Today, third‐generation synchrotrons emit collimated X‐ray beams with variable energy and spectral brightness 10 orders of magnitude higher than X‐ray tubes.^30^, ^31^, ^32^ Typically, hard X‐rays (>2000 eV) are used for characterization of (bi)metal catalysts owing to their i) high penetrating power, required for in situ and operando metrology, ii) high scattering cross‐section for metal atoms compared to lighter elements contained in hydrocarbons or the support, and iii) element specificity originating from element‐dependent X‐ray absorption edges. In contrast, soft X‐rays (<2000 eV) have limited penetrative power but show high sensitivity and element specificity towards lighter elements, for example, contained in reaction products (for example, C_*x*_H_*y*_), and metal valence‐band properties. Figure 2 provides a brief overview of X‐ray tools to study supported metal catalysts, while Figure 3 shows which X‐ray tools can be used to yield structural or electronic information at specific length scales. In what follows, the advantages and limitations of these X‐ray methods are discussed for the study of bimetallic NPs.

### 3.1 X‐ray Absorption, Emission, and Photoelectron Spectroscopy

In X‐ray absorption spectroscopy (XAS), a core‐level electron is excited to unoccupied valence states or the continuum.^33^ By varying the incident X‐ray energy across an absorption edge, the X‐ray absorption near‐edge structure (XANES) and extended X‐ray absorption fine structure (EXAFS) are recorded. XANES yields information on the unoccupied valence states and the local geometry, while EXAFS provides structural details on the local environment around the X‐ray absorber. The main advantage of XAS over other X‐ray techniques is that it does not require long‐range order and the technique is element‐specific.

The core hole generated during X‐ray absorption is filled by electrons from higher energy levels, leading to X‐ray and Auger emission. Fluorescence detection of the emitted X‐rays is advantageous compared to transmission XAS as it allows to i) detect trace amounts with high sensitivity, ii) collect site‐specific XAS based on different spin states, iii) obtain high energy resolution fluorescence detected (HERFD) XAS owing to reduced core‐hole lifetime broadening of fluorescent decay channels, and iv) probe the (un)occupied density of states and differentiate between different low‐*Z* ligands by (non‐)resonant X‐ray emission spectroscopy (XES).^34^, ^35^

When the incident X‐ray energy exceeds the binding energy of core electrons, the energy of outgoing photoelectrons can be measured by X‐ray photoelectron spectroscopy (XPS), yielding the composition, oxidation state, and electronic structure of the X‐ray absorbers. The advantage of XPS relative to other X‐ray tools is its surface sensitivity, which allows for depth profiling in a small number of monolayers. Although initially the technique required UHV conditions, major progress has been made to allow near‐ambient pressure (NAP) XPS.^36^

### 3.2 X‐ray Scattering and Diffraction

X‐ray scattering results from diffuse and/or coherent scattering, the latter known as Bragg X‐ray diffraction (XRD).^37^ While intense XRD features mainly appear in the wide‐angle X‐ray scattering (WAXS, >5°) region, diffuse scattering is manifested in both the small‐angle X‐ray scattering (SAXS, <5°) and WAXS region.^38^ Diffuse scattering originates from (sub‐)nm electronic density contrast, providing i) nm‐scale structural information on the NP shape, size, and spacing at small angles, and ii) Å‐scale structural information on the interatomic distances of X‐ray scattering atomic pairs at wide angles. From the latter, a pair distribution function (PDF) can be obtained after inverse Fourier transformation. PDF does not require long‐range order (similar to EXAFS) but displays sensitivity over longer length scales without element specificity.

To reduce undesired bulk scattering from the catalyst support, NP‐decorated planar supports can be probed by grazing‐incidence SAXS and WAXS, respectively GISAXS and GIWAXS.^39^, ^40^ By setting the incident angle close to the critical angle of the support, typically below 1°, near‐surface scattering is enhanced, which provides high sensitivity to (bi)metal NPs.

### 3.3 In Situ and Operando Metrology

To probe all layers of complexity in bimetallic nanocatalysts, in situ and operando characterization is essential to capture their structural and electronic properties under relevant conditions. Owing to their penetrative nature, hard X‐rays are powerful tools to monitor nanocatalysts under harsh conditions, which resulted in the design of dedicated high‐temperature and high‐pressure reaction cells.^41^ Soft X‐ray tools favor UHV, though major progress has been made to allow gas pressures and elevated temperatures to mimic realistic conditions. For example, time‐resolved NAP‐XPS gained prominence in operando characterization,^42^ taking advantage of small dead‐volume reaction cells^43^ and fast delay line detectors. XRS can probe soft X‐ray edges using Raman scattered hard X‐rays, allowing in situ and operando conditions.

### 3.4 Time‐ and Spatially Resolved X‐ray Characterization

In the past decades, time‐resolved in situ or operando X‐ray metrology has become possible owing to the development of i) beamline optics for time‐resolved experimentation, ii) high‐temperature and high‐pressure reaction cells (Section 3.3) and product monitoring, and iii) improved detector sensitivities and data transfer. Notable examples include Quick‐XAS,^44^ Dispersive‐XAS, and high‐energy resolution off‐resonant spectroscopy (HEROS),^45^ allowing (sub‐)second XAS data acquisition for monitoring the temporal changes in catalysts. Scattering and diffraction tools mainly benefited from improved detectors with larger 2D area to capture wider 2*θ* ranges in one snapshot, smaller pixel sizes for better pattern resolution, and higher maximum count rates for the use of more intense X‐ray beams.

Current state‐of‐the‐art X‐ray nanoscopes have spatial resolutions at best approaching 15 nm, which are still away from the nm length scale required for imaging catalytically relevant single NPs.^30^ Even though tools like microfocus XRD^46^ and coherent diffraction imaging (CDI)^47^ are promising, to date (bi)metallic nanocatalyst characterization still mainly uses bulk X‐ray tools, more and more in tandem with a posteriori data treatment. For a summary of the latest developments in spatiotemporal imaging of heterogeneous catalysts, we refer to a recent review.^30^

### 3.5 A Posteriori Data Treatment

As size and information content of datasets increase, smart, often (semi‐)automated a posteriori data treatment methods can replace classic time‐expensive and comparatively inaccurate analysis.^48^ In X‐ray spectroscopy, for example, modulation excitation spectroscopy coupled with phase sensitive detection (MES‐PSD) increases the signal sensitivity towards the active part of the catalyst, for example, the NP surface in reaction, while filtering out the spectator contribution.^49^ Wavelet‐transformed (WT) XAS can simultaneously determine the atom type (k‐space) and location (R‐space) of the X‐ray absorber's neighbors, in contrast to k‐space‐blind Fourier transformed (FT) EXAFS.^50^, ^51^ For X‐ray scattering techniques, correlation methods are applied to extract scattering fluctuations over the detector pattern angles, termed X‐ray cross‐correlation analysis (XCCA),^52^ and time, named X‐ray photon correlation spectroscopy (XPCS),^53^ yielding structural information and insight in the system dynamics, respectively. A relatively recent approach, applicable to a broad set of X‐ray methods, is machine learning, which can empirically link signal features to material properties if trained thoroughly.^54^

## 4 Bimetallic Nanocatalyst Formation

To illustrate today's status and future potential of X‐ray tools, herein, we present showcase examples which mainly focus on Pt‐based bimetallic nanocatalysts. This catalyst class can serve as archetypal example as it has been at the center of decades‐long catalysis research, especially for the development and application of X‐ray tools.

### 4.1 Birth of a Nanoparticle

**Figure 4:**
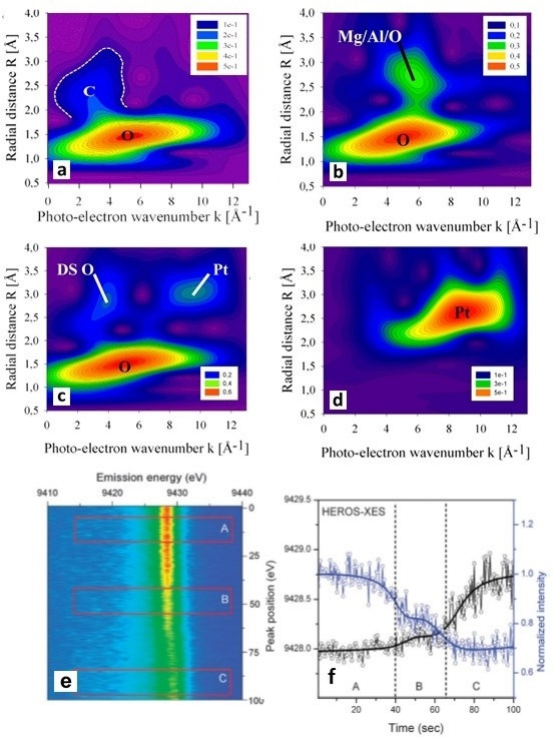
Pt L_3_‐edge WT XAS magnitude plots a) before and b) after calcination of Pt(acac)_2_/Mg(In)(Al)O_*x*,_ with c) PtO_2_ and d) Pt references. Pt L_3_ e) HEROS and f) the corresponding WL position and intensity during 150 °C H_2_ reduction of Pt(acac)_2_. Adapted from Refs. 45, 50.

In industry, impregnation methods are widely used to deposit metal precursors on porous supports, followed by drying, calcination, and reduction to yield supported NPs.^57^ Filez et al.^50^ used WT XAS to study decomposition of a Pt(acac)_2_ precursor impregnated on a Mg(In)(Al)O_*x*_ support. Prior to calcination, Pt−O bonds are observed typical of the square planar Pt(acac)_2_ geometry (Figure 4 a). The double‐scattering Pt−C−O−Pt foothill to the Pt−O peak originates from the precursor ligand geometry. After calcination at 650 °C, Pt‐Mg and/or Pt‐Al support peaks appear besides Pt−O bonds, showing a ligand change around Pt owing to acac decomposition and strong Pt–support binding during calcination (Figure 4 b). No PtO_2_ or Pt NPs are formed as their peaks are absent, which is rapidly checked by WT XAS (Figure 4 c,d), suggesting atomic Pt on the support.

Szlachetko et al.^45^ used HEROS to monitor the decomposition of Pt(acac)_2_ in H_2_ at 150 °C. By tuning the incident X‐ray energy below the Pt edge (off‐resonant), single‐shot XES can be collected by a high‐resolution dispersive spectrometer with sub‐second time resolution. XAS can then be obtained from off‐resonant XES by the Kramers–Heisenberg formalism. Self‐absorption does not occur in HEROS, opposed to fluorescence XAS. A two‐step Pt(acac)_2_ decomposition mechanism is seen in H_2_: i) a decrease of the white line (WL) height owing to a decreasing density of unoccupied 5d states, followed by ii) a peak shift showing reduction to metallic NPs (Figure 4 e,f). Notably, Saha et al.^58^ used in situ total X‐ray scattering (1 s resolution) to study Pt and Pt_3_Gd formation from their precursor states.

**Figure 5:**
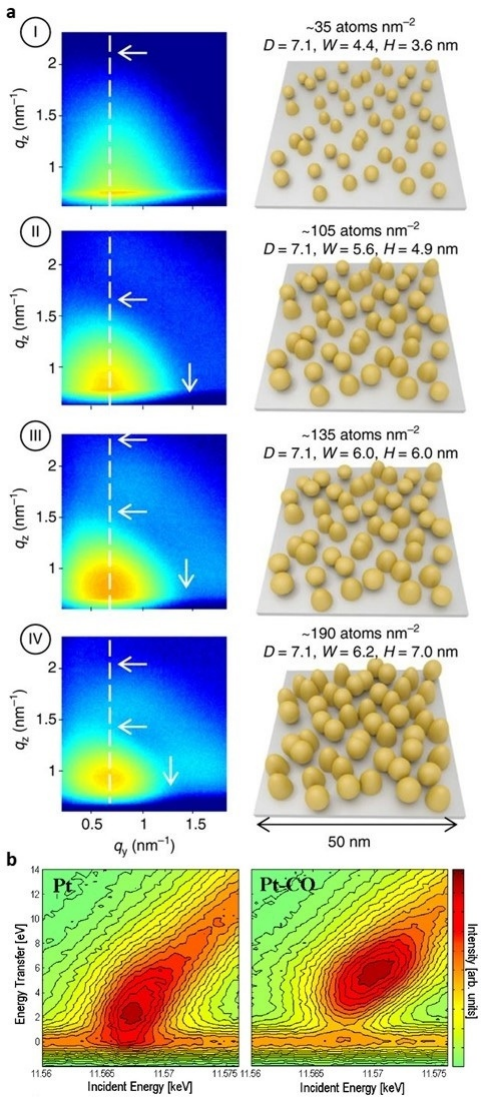
a) GISAXS patterns and overview of Pt NP morphology after 20 MeCpPtMe_3_‐O_2_ ALD cycles plus i) 0, ii) 20, iii) 30, and iv) 40 MeCpPtMe_3_‐N_2_* cycles. b) RIXS maps for bare and CO‐adsorbed Pt NPs. Adapted from Refs. 55, 56.

An emerging method to deposit metal NPs with (sub‐)nm control is atomic layer deposition (ALD).^59^ By combining O_2_ and N_2_ plasma (N_2_*) Pt ALD, Dendooven et al.^56^ independently tuned the Pt NP size and center‐to‐center distance on a planar support (Figure 5 a). GISAXS showed that after depositing a selected number of Pt NPs by MeCpPtMe_3_‐O_2_ ALD, subsequent MeCpPtMe_3_‐N_2_* ALD only increased their size, while keeping the center‐to‐center distance constant: i) the constant *q_y_*‐values of the lobe maxima evidence the constant NP spacing, while ii) the shift of the lobe minima in *q_y_* and *q_z_* to lower values and the appearance of a secondary lobe is characteristic for an increasing NP width and height, respectively. Notably, a QXAS study showed that the Pt NP size and spacing can also be tuned after synthesis by redox cycling, using a pre‐selected temperature and reduction gas, for example, H_2_ or CO.^60^ This is in line with an early Turbo‐XAS study of Nagai et al.^61^ showing temperature‐dependent in situ redispersion in Pt/CeZrYO_*x*_ automotive catalysts upon redox cycling.

While the Pt NP size and shape can be extracted from XAS^62^ and (GI)SAXS^63^ modeling, the electronic properties can be interrogated for example by RIXS.^35^ Glatzel et al.^55^ used RIXS to map the occupied density of 5d valence states of Pt_6_ NPs (by means of the energy transfer) in bare and CO‐adsorbed state (Figure 5 b). CO adsorption atop of Pt_6_ lowered the 5d band center relative to the Fermi level. The more the 5d band center moves below Fermi level, the weaker the interaction with new adsorbates;^15^ thus Pt adsorbates can change catalytic activity.

### 4.2 Alloy Formation: Hydrogen Spillover and Metal Mobility

**Figure 6:**
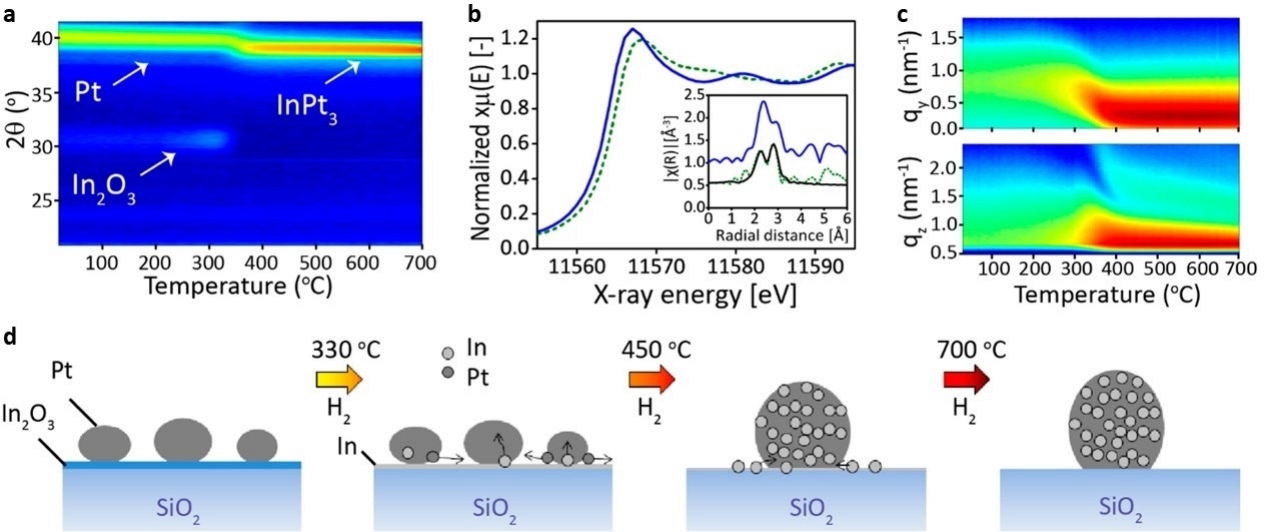
In situ a) XRD and c) horizontal (*q_y_*) and vertical (*q_z_*) GISAXS pattern cuts during H_2_‐TPR of Pt/In_2_O_3_ films. b) Pt L_3_‐edge XANES and FT EXAFS (inset) of Pt‐In alloy after H_2_‐TPR (green, dashed) and bulk Pt (blue); EXAFS model fit shown in black. d) mechanism of NP alloying. Adapted from Ref. 64.

Ramachandran et al.^64^ used XAS, in situ XRD and GISAXS to study Pt‐In nanoalloy formation starting from Pt NPs on an In_2_O_3_ support. In situ XRD during H_2_‐TPR showed a gradual Bragg shift from Pt to Pt_3_In due to In‐incorporation in face‐centered cubic (fcc) Pt (Figure 6 a). Pt_3_In alloying is confirmed by a XAS edge blue‐shift relative to bulk Pt and an In‐contribution in its fcc‐type EXAFS fingerprint (Figure 6 b). In situ GISAXS showed NP growth during Pt‐In alloying (lobe shift to smaller *q_y_* and *q_z_* values, Figure 6 c,d), with subsequent NP height increase upon further heating (secondary lobe shift to smaller *q_z_*). Filez et al.^65^, ^66^ further detailed the alloying process as follows: i) dissociation of H_2_ on Pt, ii) H spillover to and iii) long‐range transport across the support, iv) (partial) reduction of indium oxide by H, v) short‐range transport to Pt, followed by vi) full reduction to In^0^ and (vii) Pt‐In alloying.

**Figure 7:**
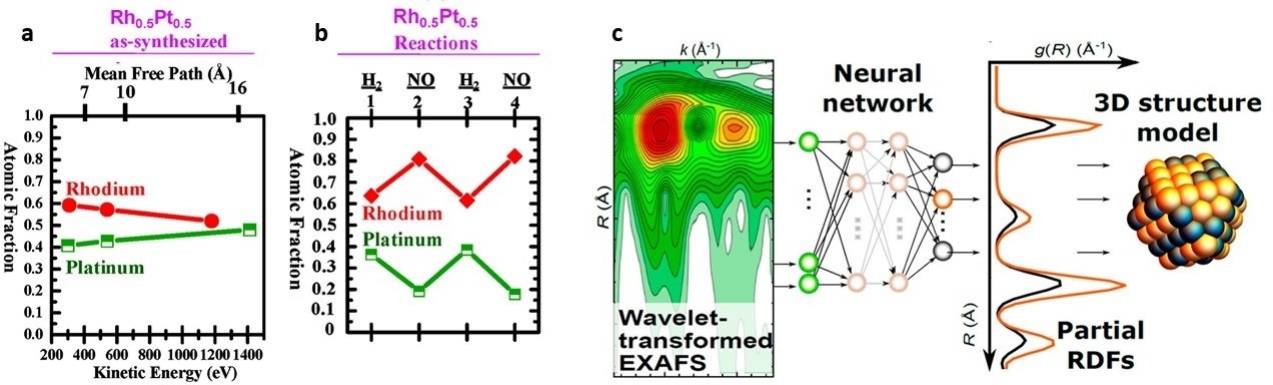
Atomic Rh and Pt fractions at the surface of Rh_0.5_Pt_0.5_ NPs as a function of a) photoelectron kinetic energy and mean free path and b) reducing and oxidizing conditions at 300 °C, obtained by AP‐XPS. c) neural network approach to derive the 3D NP structure based on WT EXAFS. Adapted from Refs. 67, 68.

Once formed, determining the size, shape, and composition of bimetallic NPs is crucial to link structural features to catalytic performance. Tao et al.^67^ used AP‐XPS to study the extent of metal mixing and segregation in bimetallic NPs under different gas atmospheres. By measuring at different incident X‐ray energies, depth profiling is achieved, showing that Rh_0.5_Pt_0.5_ alloyed NPs have good mixing properties but expose more Rh at the NP surface in vacuum (Figure 7 a). Under 100 mTorr reducing H_2_ or oxidizing NO gas, the NP surface composition changes, resulting in increased Rh segregation (Figure 7 b). Timoshenko et al.^68^ recently developed a powerful approach to reconstruct 3D atomic models of mono‐ and bimetallic NPs by combining WT XAS, supervised machine learning, and molecular dynamics simulations (Figure 7 c). Deriving the 3D NP structure in real‐time from in situ or operando XAS could revolutionize nanocatalysis.

## 5 Bimetallic Nanocatalyst Functioning

### 5.1 Active State

**Figure 8:**
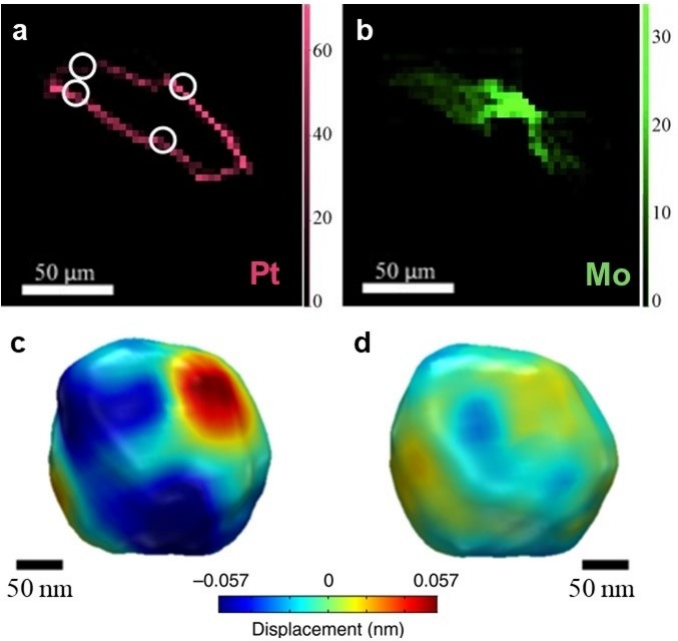
a),b) μ‐XRF‐CT cross‐section of a single Mo‐promoted Pt/C catalyst particle, showing a) Pt and b) Mo elemental maps during liquid‐phase hydrogenation of nitrobenzene. c),d) Bragg CDI‐reconstructed 3D images showing the displacement distribution (lattice distortion) on a single 220 nm Pt NP at the c) start and d) end of methane oxidation. Adapted from Refs. 47, 69.

X‐ray microscopy shows potential to map the nanocatalyst active state under reaction or relevant conditions. Using microfocus scanning XRF combined with computed tomography (μ‐XRF‐CT), Price et al.^69^ mapped the Pt and Mo composition across a Mo‐promoted Pt/C catalyst particle during liquid‐phase hydrogenation of nitrobenzene with 5×5 μm^2^ pixel size. Strong intraparticle heterogeneities were observed, namely Mo residing mostly in the particle core while Pt is strongly abundant at the edge (Figure 8 a,b). In contrast to the μm resolution of μ‐XRF‐CT, Bragg coherent diffraction imaging (CDI) can map in situ the lattice displacement in a model Pt NP during methane oxidation with 14 nm resolution.^47^ Such displacements originate from reactant adsorption and thus allow for active site localization. Strong distortions are observed at the NP surface corner and edge regions owing to methane oxidation, which are restored after reaction (Figure 8 c,d).

### 5.2 Dynamic Restructuring

**Figure 9:**
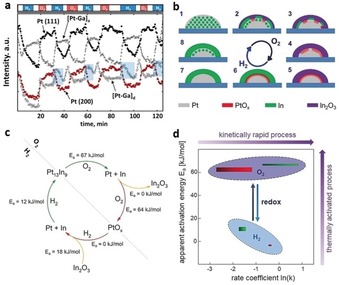
a) integrated XRD peak intensities (Pt(111), Pt(200), Pt‐rich [Pt‐Ga]_a_, Pt‐poor [Pt‐Ga]_d_) vs. H_2_‐He‐O_2_‐He redox time at 600 °C. Blue‐shaded areas indicate transient Pt‐poor [Pt‐Ga]_d_ phase evolution. b) Mechanism of reversible Pt‐In alloying‐segregation at the single H_2_–O_2_ cycle level: (1–5) simultaneous Pt_13_In_9_ segregation, Pt surface oxidation, (5–8,1) consecutive Pt reduction and Pt‐In alloying. c) reaction mechanism including Arrhenius parameters as derived from QXAS kinetic modelling. d) *E*_a_−ln(*k*) plot for (red, top) Pt oxidation, (green, top) Pt‐In segregation in O_2_ and (green, bottom) Pt‐In alloying and (red, bottom) PtO_*x*_ reduction in H_2_. The rectangle width represents the range to which ln (*k*) varies over the measured temperature range; its height is the 66 % confidence interval of *E*_a._ Adapted from Refs. 70, 71.

Redekop et al.^70^ investigated the dynamic phase changes in activated Mg(Ga)(Al)O_*x*_‐supported Pt‐Ga nanoalloys during 600 °C H_2_–O_2_ redox cycling (Figure 9 a). Such conditions mimic reaction–regeneration cycles in the industrial propane dehydrogenation processes (for example, Oleflex) or rapid redox cycles in car exhaust convertors. After one redox cycle, H_2_ gas leads to Ga_2_O_3_/Pt reduction causing Pt‐Ga nanoalloy formation into a Pt‐rich intermetallic or solid solution, and a Ga‐rich phase which disappears over longer timescales, presumably a transient NP surface alloy, which might affect the catalyst performance.

Recently, Filez et al.^71^ refined the steps involved in segregation alloying of Pt_13_In_9_ into In_2_O_3_/PtO_*x*_ and back to Pt_13_In_9_ during high‐temperature O_2_–H_2_ redox cycling (Figure 9 b). By kinetic modeling of QXANES data, partial reaction orders, rate constants, and Arrhenius parameters were estimated, allowing to construct the kinetic reaction cycle that steers the dynamic restructuring of Pt_13_In_9_ nanoalloys (Figure 9 c). In O_2_, Pt_13_In_9_ decomposition and Pt surface oxidation simultaneously take place with high activation energy in which Pt oxidation is rate‐determining (Figure 9 d). In contrast, the reverse processes in H_2_ steer the equilibrium state from In_2_O_3_/PtO_*x*_ back to Pt_13_In_9_ through In_2_O_3_ and PtO_*x*_ reduction followed by Pt‐In alloying, both showing low apparent activation energies. Kinetic modeling of QXAS can thus identify the reaction steps governing the dynamic restructuring of the nanocatalyst.

Beyond model redox cycling, Virnovskaia et al.^72^ found with AP‐XPS that the surface of Pt‐Sn nanocatalysts is Sn‐enriched during high‐temperature alkane dehydrogenation and Sn remains partially oxidized even in a highly reducing hydrocarbon environment. Barbosa et al.^73^ showed by AP‐XPS during methanol steam reforming that the surface of Pt‐In NPs is In‐enriched, suppressing CO formation during reaction.

### 5.3 Deactivation by NP Sintering

**Figure 10:**
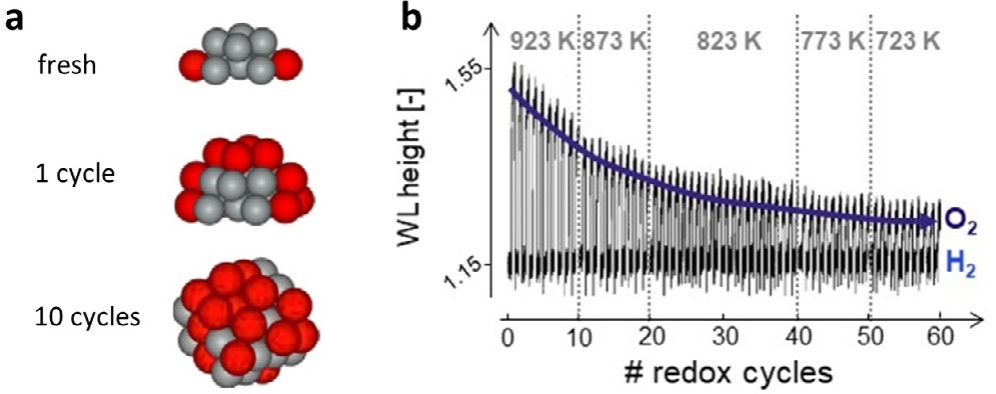
a) simulated Pt‐Sn NP morphologies for the fresh, 1× and 10× [C_3_H_8_‐O_2_‐H_2_] cycled catalyst. b) XANES WL height evolution during O_2_–H_2_ redox cycling of a Pt‐In nanocatalyst. Adapted from Ref. 71, 74.

During NP sintering, the number of active sites decreases by NP growth. Iglesias‐Juez et al.^74^ simulated possible Pt‐Sn NP structures based on Pt‐Pt and Pt‐Sn EXAFS coordination numbers^75^ for different dehydrogenation‐regeneration C_3_H_8_–O_2_–H_2_ cycle(s), showing: 1) NP size increase, 2) progressive Sn‐enrichment of the NP, and 3) increased mixing from core–shell‐ to random‐type alloys (Figure 10 a). A gradual NP size increase is also observed by Filez et al.^71^ via a decreased XANES WL height under O_2_ during 60 H_2_–O_2_ redox cycles (Figure 10 b). In O_2_, an In_2_O_3_/PtO_x_ composite is formed in which PtO_*x*_ consists of a metallic core with oxidized surface, the latter yielding increased WL heights. With decreasing dispersion, which is due to sintering during redox cycling, the fraction of oxidized surface decreases, leading to a WL decrease. Notably, Solano et al.^76^ also studied Pt NP sintering with in situ GISAXS under different O_2_ partial pressures to monitor the NP size and spacing in real time. The observed behavior indicates the key role of the outer PtO_2_ shell, stable at low temperature, and its thermal reduction creating mobile species that trigger particle sintering.

## 6 Conclusions and Outlook

A complementary X‐ray toolbox is currently available to extract the size, shape, composition, spacing, and electronic properties of bimetallic NPs under in situ or operando conditions. These tools include X‐ray absorption, emission, and photoelectron spectroscopy along with scattering and diffraction, often in combination with advanced a posteriori data treatment. This toolbox currently provides mechanistic insights into the formation, functioning, and deactivation of nanocatalysts in real time, leading to a more detailed understanding of bimetallic NPs.

Future trends will favor increased spatiotemporal resolution of the X‐ray experiment. On the one hand, X‐ray techniques allowing milli‐ to femtosecond time resolution have strong potential to uncover currently scarce kinetic descriptions of transient changes in catalytic solids and molecular adsorption–desorption phenomena under reaction conditions, respectively. On the other hand, the avenue of spatially resolved X‐ray micro‐ and nanoscopy will provide the means to map the composition and structure of metal catalyst particles and single crystals under real conditions, for example by (S)TXM,^77^, ^78^ μ‐XRD‐CT,^46^ or CDI,^47^ uncovering particle heterogeneities commonly present in heterogeneous catalysts.^79^, ^80^ As the spatial resolution of state‐of‐the‐art X‐ray nanoscopes (ca. 15 nm) still exceeds the dimensions of catalytically relevant NPs, machine learning methods for obtaining the 3D NP structure show disruptive potential to revolutionize this field of research.^30^, ^68^ Methods which increase the sensitivity of a technique, such as wavelet analysis^50^ or MES,^49^ can be used to efficiently extract apparently hidden information.

Future advances greatly rely on new X‐ray sources. The advent of X‐ray free‐electron lasers (XFELs) announces highly coherent, intense, and short‐pulsed X‐ray beams, facilitating the investigation of matter dynamics on atomic length scales with femtosecond time resolution.^81^ Furthermore, laboratory‐based XAS, XES, and (GI)SAXS become increasingly popular, allowing everyday access to traditional synchrotron techniques.^82^ These evolutions hold bright prospects for X‐ray tools in nanocatalysis, and more general in functional materials research.

## Conflict of interest

The authors declare no conflict of interest.

## Biographical Information

*Matthias Filez studied Chemical Engineering and Physics at KU Leuven. He received his PhD in 2015 at Ghent University with Prof. Guy B. Marin and Prof. Christophe Detavernier on the “Alternative Design of Pt‐based Catalysts: An X‐ray Spectroscopic View”. He is currently a Marie Skłodowska‐Curie postdoctoral researcher at Utrecht University*.

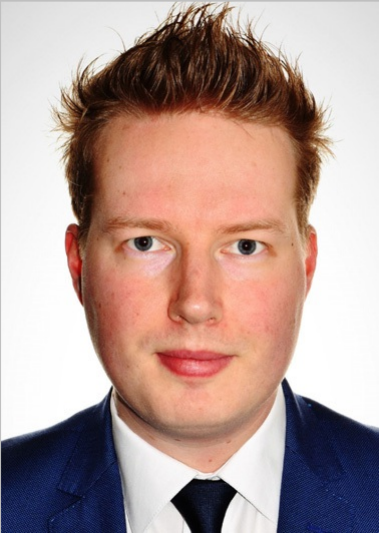

## Biographical Information

*Vladimir V. Galvita received his PhD in Chemistry in 1999 at Boreskov Institute of Catalysis*, *after which he continued as postdoctoral researcher at Max‐Planck‐Institute and University of California, Berkeley. He is a Professor in Chemical Process Technology at Ghent University. His research is focused on understanding structure‐composition‐performance relationships in heterogeneous catalysts*.

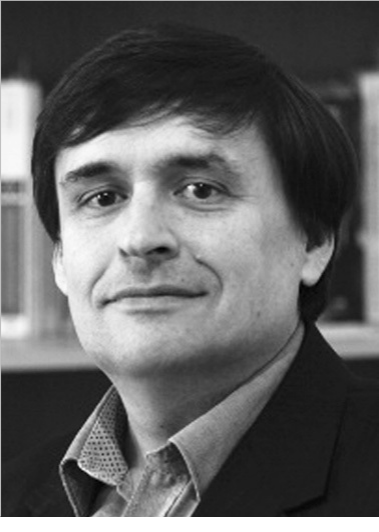

## Biographical Information

*Christophe Detavernier earned his PhD in Physics at Ghent University. Following a postdoctoral position at IBM's T.J. Watson Research Center, he joined the Department of Solid State Sciences at Ghent University as the Director of the Conformal Coatings for Nanomaterials group in 2005. His research interests include atomic layer deposition and in situ characterization of thin films*.

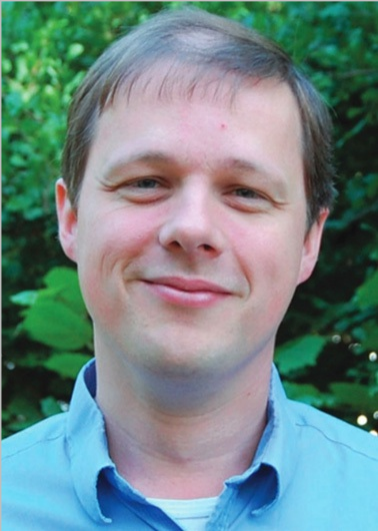

## Biographical Information

*Guy B. Marin received his PhD in Chemical Engineering at Ghent University in 1980*, *after which he continued as postdoctoral researcher with Prof. M. Boudart at Stanford University. He became Professor in Chemical Technology at Eindhoven University of Technology, and subsequently Director of the Laboratory for Chemical Technology at Ghent University*.